# Cognitive load decreases cooperation and moral punishment in a Prisoner’s Dilemma game with punishment option

**DOI:** 10.1038/s41598-021-04217-4

**Published:** 2021-12-30

**Authors:** Laura Mieth, Axel Buchner, Raoul Bell

**Affiliations:** Department of Experimental Psychology, Heinrich Heine University Düsseldorf, Universitätsstrasse 1, 40225 Düsseldorf, Germany

**Keywords:** Psychology, Human behaviour

## Abstract

The present study serves to test whether cooperation and moral punishment are affected by cognitive load. Dual-process theories postulate that moral behavior is intuitive which leads to the prediction that cooperation and moral punishment should remain unaffected or may even increase when cognitive load is induced by a secondary task. However, it has also been proposed that cognitive control and deliberation are necessary to choose an economically costly but morally justified option. A third perspective implies that the effects of cognitive load may depend on the specific processes involved in social dilemmas. In the present study, participants played a simultaneous Prisoner’s Dilemma game with a punishment option. First, both players decided to cooperate or defect. Then they had the opportunity to punish the partners. In the cognitive-load group, cognitive load was induced by a continuous tone classification task while the no-load group had no distractor task. Under cognitive load, cooperation and moral punishment decreased in comparison to the no-load condition. By contrast, hypocritical and antisocial punishment were not influenced by the dual-task manipulation. Increased cognitive load was associated with a bias to punish the partners irrespective of the outcome of the Prisoner’s Dilemma game, suggesting that punishment was applied less purposefully in the cognitive-load condition. The present findings are thus in line with the idea that the availability of cognitive resources does not always have a suppressive effect on moral behaviors, but can have facilitating effects on cooperation and moral punishment.

## Introduction

Are humans intuitively selfish and only able to suppress their selfish impulses by deliberately controlling their behavior? Or are humans intuitively cooperative and selfish behavior is the result of cold rationality overriding the moral intuitions? This issue has received considerable attention and the answer to this question has changed over time. Traditionally, the dominant view in economics and biology was that humans are predisposed to maximize their own selfish gain so that selfish impulses have to be controlled by reason (for reviews, see^1,2^). However, this view has been challenged. On the basis of dual-process theories^3^, it has been postulated that cooperation is intuitive^4,5^. Therefore, decisions in moral dilemmas should be more cooperative and less selfish when cognitive resources are scarce, for example, when attention is divided between two tasks. However, evidence concerning how cooperation and moral behavior is affected by the availability of cognitive resources is mixed (e.g.^6–11^), suggesting that the effect of cognitive resources on cooperation may depend on the specific processes involved in a certain task^2,12^. Here, we revisit this issue and extend previous studies by focusing on moral punishment of non-cooperative behavior. Including punishment in this discussion is interesting because moral punishment promotes cooperation by removing the incentives for selfish behaviors^13,14^. Furthermore, moral punishment in one-shot interactions can be seen as a form of moral behavior because it is costly and has no immediate benefits for those who punish^15^. It thus seems interesting to examine to what extent moral punishment is intuitive or deliberate.

**Figure 1:**
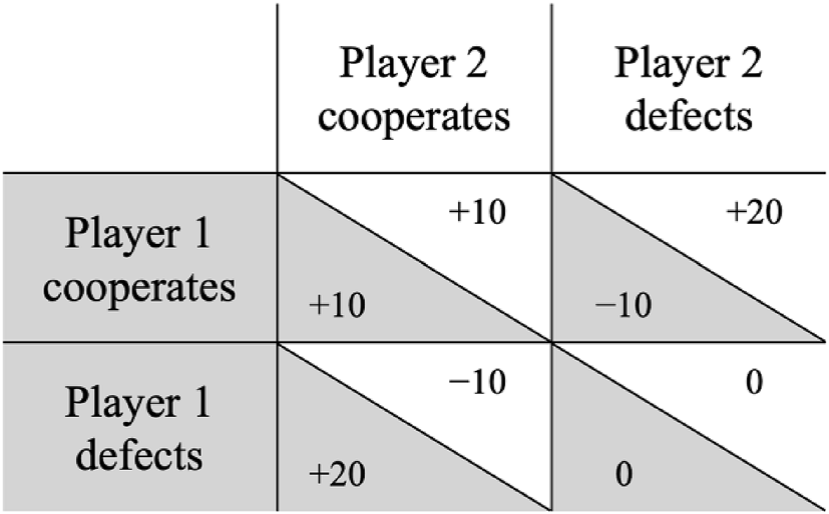
An example for the payoff matrix of a Prisoner’s Dilemma game. Values on gray backgrounds represent the payoffs of Player 1 while values on white backgrounds represent the payoffs of Player 2. In the Prisoner’s Dilemma game, unilateral defection yields the highest payoff, followed by mutual cooperation which, in turn, yields a higher payoff than mutual defection. Unilateral cooperation yields the lowest payoff. Each player benefits from choosing defection, regardless of what the other player does. This introduces an incentive for defection for each individual. However, when both players follow their selfish interests, they are collectively worse off than when they cooperate with each other.

Cooperation is important for human groups and societies because it increases the chances for survival and development. However, cooperation often involves accepting costs for the benefit of others^16^. In these situations, cooperation represents a moral dilemma because there is a conflict between the individual’s selfish interest and what is collectively best. This creates a free-rider problem because there is an incentive to cheat: free-riders have an advantage by accepting the help of others and by shying away from the costs of reciprocating^17^. This conflict between collective and individual interests is illustrated in the Prisoner’s Dilemma^18^. In the classical Prisoner’s Dilemma game, two players have to decide simultaneously whether they want to cooperate or defect. A possible payoff matrix for the individual players is displayed in Fig. 1. For the individual, the best possible outcome is achieved via unilateral defection, while the worst possible outcome results from unilateral cooperation. However, for both players collectively, mutual cooperation leads to a better outcome than mutual defection. This illustrates that, at a collective level, cooperation is desirable because common goals can be better achieved by working together. However, each individual is better off when defecting. The only Nash equilibrium^19^ of this game therefore is that both players choose to defect.

Despite these negative conditions, cooperative individuals are able to achieve high levels of cooperation in social-dilemma situations^20^. However, free riders may try to exploit these cooperative tendencies. Free riders cheat by benefitting from the cooperation of others while failing to reciprocate anything in return. To maintain high levels of cooperation, it is thus necessary to solve the free-rider problem by excluding free-riders from cooperative exchange or by punishing them. Punishment is a solution to the free-rider problem because it imposes costs on free-riders that remove the incentives of cheating^20,21^. When cheating is consistently punished, the rate of cheating will therefore decrease. In principle, the fear of punishment can allow for cooperation to emerge even in situations in which cooperation may seem unlikely, for example, in one-shot interactions in which people interact with their partners only once so that there is a strong incentive to cheat because there is a low chance of reciprocation. Punishment can thus be seen as a moral behavior that is efficient in promoting cooperation. However, punishment in one-shot interactions is costly and does not provide direct benefits to those who punish. While the group as a whole may benefit from moral punishment, economic rationality dictates that each individual should shy away from the costs of punishing others. The moral punishment of non-cooperative behavior can thus be seen as a form of second-order cooperation^22^.

During the last decade it has become increasingly popular to consider cooperation through the lens of dual-process theories^3^. In general, dual-process theories imply that human behavior can be understood as resulting from the interplay of two fundamental modes of processing^23^. The first mode is characterized by intuitive, heuristic and effortless processing (Type I) that is fast and automatic. The second mode is characterized by deliberate, analytical and effortful processing (Type II) that is slower and controlled. When cognitive resources are available, Type-II processing can override Type-I processing (e.g., when deliberately deciding to eat a healthy apple instead of a delicious piece of cake to keep one’s diet goals). When cognitive load is imposed by a secondary task or when time constraints are implemented, behavior is assumed to shift from the deliberate mode to the intuitive mode of thinking.

There is an ongoing debate on whether moral behaviors are intuitive or deliberate. A common view is that people are only able to make the moral choice to help others when they exert deliberate control over their selfish impulses (e.g.^24^). This *deliberate-morality view* echoes the classical idea that humans have selfish impulses that have to be kept under control by reason (for reviews, see^1,2^). However, this view has lost popularity over the last decade. In two influential publications, Rand et al.^4,5^ provided evidence that cooperation is intuitive. Participants played a one-shot Public Goods game. The Public Goods game can be seen as a generalization of the Prisoner’s Dilemma game in which multiple group members decide, at the same time, whether to invest into the game^25^. Rand and colleagues found that participants cooperated more when time pressure was imposed on the participants than when their responses were delayed. This was true even though the interactions were one-shot and thus did not favour reciprocal cooperation. It was concluded that cooperation in one-shot interactions with monetary stakes is an intuitive process while deliberation leads to less cooperative and economically more rational behavior. This research led to the formulation of the *intuitive-morality view*^1^, according to which “intuition supports cooperation in social dilemmas, and that reflection can undermine these cooperative impulses”^4,^. These conclusions were supported by results showing that cognitive load, induced via a secondary task, increases cooperation^8,9,11,26^. However, these broad conclusions about the cognitive basis of cooperation were challenged by follow-up studies that did not find evidence of an influence of cognitive load or time pressure on cooperation^6,27^ and by studies that found cognitive load and time pressure to be associated with decreased cooperation rates^28^. The mixed evidence on the influence of cognitive load and time pressure on cooperation suggests that whether cooperation is intuitive or reflective depends on other factors (e.g.^2^). For example, certain situational aspects may change the focus of intuitive and reflective decisions^12^. One of these factors may be the presence of moral punishment. When people are punished for failing to cooperate, this changes the incentive structures of moral dilemmas. When moral punishment is consistently applied, cooperation becomes the economically rational choice. It then follows that deliberation should favor cooperation under these circumstances (cf.^5^).

The main purpose of the present study was to test whether the probability of moral punishment increases or decreases under cognitive load. Given that moral punishment is described as a form of second-order cooperation^22^, it seems plausible that moral retributive punishment could be seen as intuitive behavior according to the intuitive-morality view^5^ and should require few cognitive resources. However, at an empirical level the link between cooperation and punishment is not straightforward^29^ and the evidence on the effects of cognitive load and time pressure on punishment is mixed^30–35^. Most of the evidence comes from the Ultimatum game. In the Ultimatum game^36^, a proposer receives a certain amount of money that they can arbitrarily split between themselves and a responder. The responder either accepts or rejects the offer. If the offer is accepted, both partners get the share that was determined by the proposer. If the offer is rejected, none of the partners gets anything. From an economically rational point of view, responders should accept even very small offers that are extremely unfair. However, unfair offers are frequently rejected. Given that the responders are willing to forgo monetary gains to reject violations of the fairness norm, their behavior is often interpreted as a form of costly punishment^37^. It then follows from the intuitive-morality view that the rejection rate should increase under cognitive load and time pressure. While some findings are consistent with this hypothesis (e.g.^30^), others are inconsistent or contradictory (e.g.^32,34^). Furthermore, it has been found that the disruption of brain structures that are associated with cognitive control decreases the rejection rate^38^. A potential concern when interpreting these findings is that cooperation and punishment are not clearly separated in the Ultimatum game. A responder may reject an offer because they want to punish the proposer for being unfair. However, the rejection of an offer may also be interpreted as a rejection of cooperation. Therefore, the degree to which rejection in the Ultimatum Game can be seen as costly punishment remains controversial (see also^39^). It thus seems interesting to examine the effect of cognitive load on costly punishment in a paradigm in which cooperation and punishment can be clearly separated from each other.

In the present study, we measure cooperation and punishment in a one-shot Prisoner’s Dilemma game with a subsequent punishment option that does not only allow to clearly separate cooperation and punishment, but also allows us to distinguish between different types of punishment^40–42^. *Moral punishment* refers to the punishment of uncooperative partners by cooperative individuals. This form of punishment is most commonly observed in social-dilemma games and helps to establish a cooperation norm^13,22,43^. Given that moral punishment is individually costly but collectively beneficial, it can be viewed as a form of second-order cooperation^22^. Based on the intuitive-morality view, moral punishment should increase under cognitive load and should decrease when cognitive resources enable greater deliberation, allowing participants to make more rational choices. However, it can also be argued that moral punishment requires cognitive control. For instance, Declerck and Boone^2^ reviewed evidence suggesting that brain structures associated with cognitive control and deliberation are consistently involved in punishment decisions precisely because there is a conflict between economic and moral aims, and speculate that it is possible that in some circumstances “self control [may be] necessary to overcome the economic cost of punishing and abide by the collectively beneficial norm” (p. 139).

Even though the intuitive-morality view only refers to moral behaviors, the picture is incomplete without recognizing that there are other types of punishment that are not necessarily based on moral motives. Based on the idea that the intuitive or deliberate nature of social behaviors depends on the specific processes involved^2^, cognitive load can be expected to have a differential effect on these types of punishment. *Hypocritical punishment* is applied when a defecting participant punishes a defecting partner. Hypocritical punishment after mutual defection is less prevalent than moral punishment but occurs with an appreciable rate. It is often attributed to spitefulness^43,44^, but may also be interpreted as a truly hypocritical retaliation of the partner’s defection^42^. *Antisocial punishment* occurs when a defector punishes a cooperating partner. Antisocial punishment is reliably observed in social dilemmas with punishment option^45–48^. This type of punishment can be interpreted as an aggressive act aimed at hurting the partner. In the cases of hypocritical and antisocial punishment, participants are thus willing to accept monetary costs to display spiteful and aggressive behavior. It thus seems questionable whether deliberation should increase these behaviors that are neither economically rational nor morally desirable. Finally, it seems necessary to take into account that punishment may occur randomly, that is, without being clearly associated with the partner’s behavior^42^. For example, it seems possible that participants are generally more or less willing to use the punishment option when cognitive load is induced. It therefore is important to distinguish an unspecific punishment bias from the other types of punishment that are specifically triggered by the partner’s behavior. If a purposeful application of punishment requires cognitive resources, more unsystematic behavior may emerge when these resources are lacking, which implies that the punishment bias should increase when cognitive resources are exhausted by cognitive load.

## Method

### Participants

The data of 206 participants (125 female) with a mean age of 23 (*SD* = 4) were analyzed. Upon arrival at the laboratory, participants were alternately assigned to the no-load condition or the cognitive-load condition. Data sets of four participants in the cognitive-load condition were excluded prior to data analysis because they had identified less than 75% of the tones in the continuous tone classification task correctly (note, however, that the conclusions remain the same when the data sets are included into the analyses). This resulted in *n* = 105 data sets in the no-load condition and *n* = 101 data sets in the cognitive-load condition. With this sample size, 14 rounds of the Prisoner’s Dilemma game and α = 0.05, small effects of the size *w* = 0.07^49^ could be detected with a statistical power of 1 − β = 0.95 when comparing the cooperation and punishment parameters between the no-load condition and the cognitive-load condition^50^. After the experiment was completed, all participants received the money shown in their final account balance (participants knew from the beginning that they played for real money) as well as course credit or a monetary compensation for participation.

### Ethics

All participants gave written informed consent in accordance with the Declaration of Helsinki. The experiment was approved by the ethics committee of the Faculty of Mathematics and Natural Sciences of Heinrich Heine University Düsseldorf. At the end of the experiment participants were debriefed that they had interacted with computer-controlled partners and were reminded that they had the opportunity to withdraw their consent to using their data (none of the participants, however, decided to do so).

### Materials and procedure

**Figure 2:**
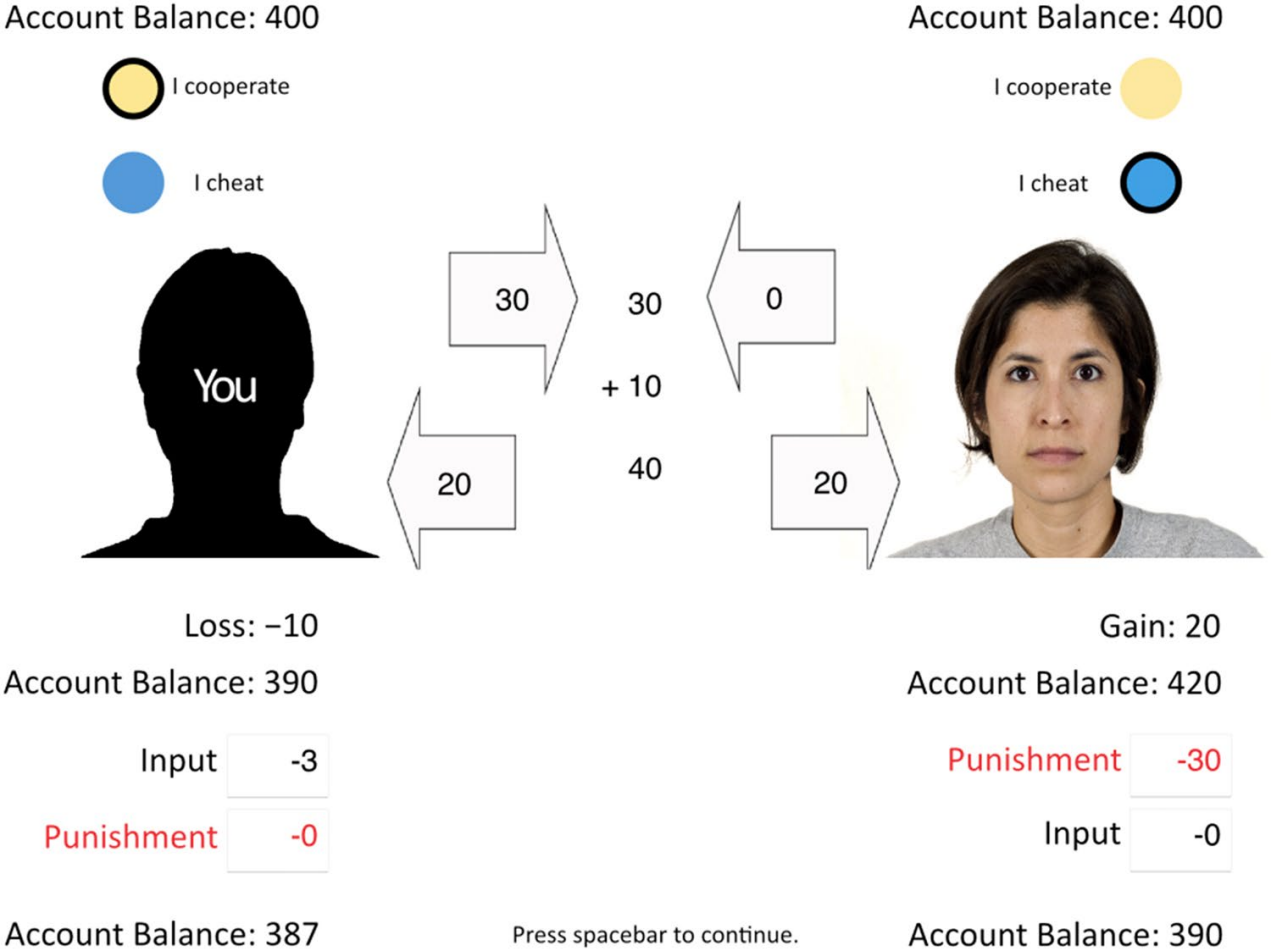
A screenshot of the simultaneous Prisoner’s Dilemma game with costly punishment option (translated version; the version used in the experiment was in German). Participants chose to cooperate or to defect in the Prisoner’s Dilemma game by pressing the up or down arrow key that were marked yellow and blue, respectively. In the example displayed here, the participant on the left cooperates while the partner on the right defects. The participant then invests 3 cents to punish the partner by reducing her account balance by 30 cents. The facial photograph on the right side was taken from the Chicago Face Database^51^.

Participants played a simultaneous Prisoner’s Dilemma game with a costly punishment option, as shown in Fig. 2. This paradigm has been extensively validated in previous studies^40–42^ in which it has been demonstrated that the participants respond to social cues of their partners’ faces^40,41^ and the moral framing of the game^42^. Participants played 20 trials with 20 different partners, a randomly selected half of which cooperated and the other half cheated. This implies that every participant experienced exactly 10 cooperating and 10 defecting partners. The first three interactions with each type of partner (cooperators, defectors) served as training trials and were not included in the analysis. The trials were presented in a random order.

At the beginning of the experiment, each participant was endowed with 400 cents that they could invest in the Prisoner’s Dilemma game. All of the transactions in the game were made in cents which has the advantage that they are easy to process given that cents is a currency in which transactions are made on a daily basis. The participant was not given elaborative deceptive instructions about the human nature of their partners, but was also not explicitly informed that they would play with computer-controlled partners before the experiment either. Instead, the instructions focused on the different outcomes that could result from the Prisoner’s Dilemma game. The participant was informed that they played with real money and that they would receive the money in their account at the end of the experiment.

### Prisoner’s Dilemma Game

The participant started each trial by pressing the spacebar. After 3 s, the participant saw a silhouette on the left side of the screen representing themselves. On the right side of the screen, a color photograph of a partner (333 × 250 pixels) was shown to emphasize the social nature of the game and to make the game more accessible to the participants. The interactions were one-shot so that the participant played with each of the 20 partners only once. In each trial, the photograph of the partner’s face was randomly selected from a pool of 90 female or 90 male faces of young white adults from the Chicago Face Database^51^ with the restriction that the participant always played with a partner of their own gender. The face of the partner was shown from a frontal view and had a neutral facial expression. The participant’s account balance was displayed above the participant’s silhouette. The partner’s account balance (displayed above the partner’s face) at the start of each trial was at most 10 cents above or below the account balance of the participant.

The participant chose “I cooperate” or “I cheat” by clicking on the color-coded arrow keys on the keyboard. The participant’s choice was highlighted with a black frame. The partner’s choice was revealed at the same time. If the participant and the partner chose to cooperate, this meant that they each invested 30 cents into the game. To defect meant to refuse to invest anything. The investments were displayed in arrows for 750 ms, after which these arrows moved towards the center of the screen (within 750 ms). The sum of investments was displayed at the center of the screen. After 750 ms, the bonus was displayed. This bonus was always 1/3 of the sum of investments and served as an incentive for cooperation. The bonus was added to the sum of investments. After 750 ms, the total sum was displayed. Each of the two players received half of the total sum, regardless of their contribution. After 750 ms, each player’s share was displayed in an arrow that moved from the center of the screen to each respective player (within 750 ms). After 750 ms, it was displayed how much money the two players had gained or lost due to the interaction. Then the updated account balances of the participant and the partner were displayed below the participant’s silhouette and the partner’s face, respectively. Mutual cooperation thus resulted in a gain of 10 cents for each player while mutual defection resulted in neither a gain nor a loss. Unilateral cooperation, however, resulted in a loss of 10 cents for the cooperating player while the defecting player gained 20 cents. This implies that the payoff structure of the game corresponded to that of a typical Prisoner’s Dilemma game in which there is a conflict between collective and individual interests^18^.

### Punishment

The punishment options for both the participant and the partner was shown 750 ms after the current trial in the Prisoner’s Dilemma game had been completed. The participant used the number keypad to select how much money they wanted to invest to punish their partner. The participant invested 0 cents if they did not want to punish their partner or 1 to 9 cents if they wanted to punish the partner. For each cent the participant invested, 10 cents were subtracted from the partner’s account. This punishment ratio implies that punishment can be effectively used to reduce the unfair payoff imbalance that results from the interaction with a cheating partner. The specific punishment ratio (invest 1 cent to deduct 10 cents from the partner’s payoff) has the additional advantage that it is particularly easy to understand. As soon as participants typed their investment into the text field below their silhouette, the punishment of the partner was displayed in the text field below the partner’s face. The participants were informed that the partner’s decision to punish them was made simultaneously with their own decision to punish the partner, but the partner’s decision was revealed 750 ms later to allow the participants to process each piece of information one by one. Following the unilateral defection of the participant, the partner invested a random amount between 1 and 9 cents to subtract 10 to 90 cents from the participant’s account balance. If the participant had cooperated or the partner had defected, no punishment was applied by the partner. This mimics the behavior of real participants who primarily use the punishment option to punish unilateral defection^40–42^. After 1 s, the updated account balances of the two players were shown at the bottom of the screen. By pressing the spacebar, participants continued to a page from which they could start the next trial.

### Cognitive load manipulation

Upon arrival, participants were alternately assigned to either the no-load group or the cognitive-load group. Participants in the no-load condition played the game without having to perform another task.

Participants in the cognitive-load condition played the exact same game but also had to perform a tone classification task which served to induce cognitive load. Continuous tone classification tasks are routinely used to examine whether the processes underlying certain behaviors depend on cognitive resources (e.g.^52,53^). The continuous tone classification task has the additional advantage that it has been empirically validated. Specifically, the task disrupts both verbal and spatial working memory and thus is effective in blocking domain-general cognitive resources^10^. In response to the piano tones C1, F3 and B6, the participant was required to press “A”, “S” and “D” on the computer keyboard that were marked black, grey and white, respectively. Each tone lasted for one second. The tones were played continuously via the headphones with high-insulation hearing protection covers (beyerdynamic DT-150) that all participants wore throughout the whole experiment. A tone was continuously repeated every second until a response was made, after which a different tone was played and repeated until it was classified. The tones were randomly selected for presentation with the restriction that a tone that had just been presented was not immediately repeated after a response had been made.

The continuous tone classification task was explained after the participant had received the instructions for the Prisoner’s Dilemma game. A training phase followed which served to familiarize participants with the continuous tone classification task. The participant had to make 20 correct tone classifications in a row to demonstrate that they had understood the continuous tone classification task and was able to master it. The participant was then informed that they had to play the Prisoner’s Dilemma game and perform the tone classification task at the same time. The participant was instructed that the tone classification should be performed without breaks, as fast as possible and with as few errors as possible.

In the experiment proper, the continuous tone classification task started immediately after the participant had hit the space bar to start a game trial. Three seconds later the Prisoner’s Dilemma game began. After each trial had been completed, the participant received a performance feedback about the percentage of tones that had been correctly classified and their mean response time in the continuous tone classification task. On average, participants classified 92 percent (*SD* = 5) of the tones in the experiment correctly and made those responses within 1560 ms (*SD* = 397).

### Measuring cooperation and punishment

**Figure 3:**
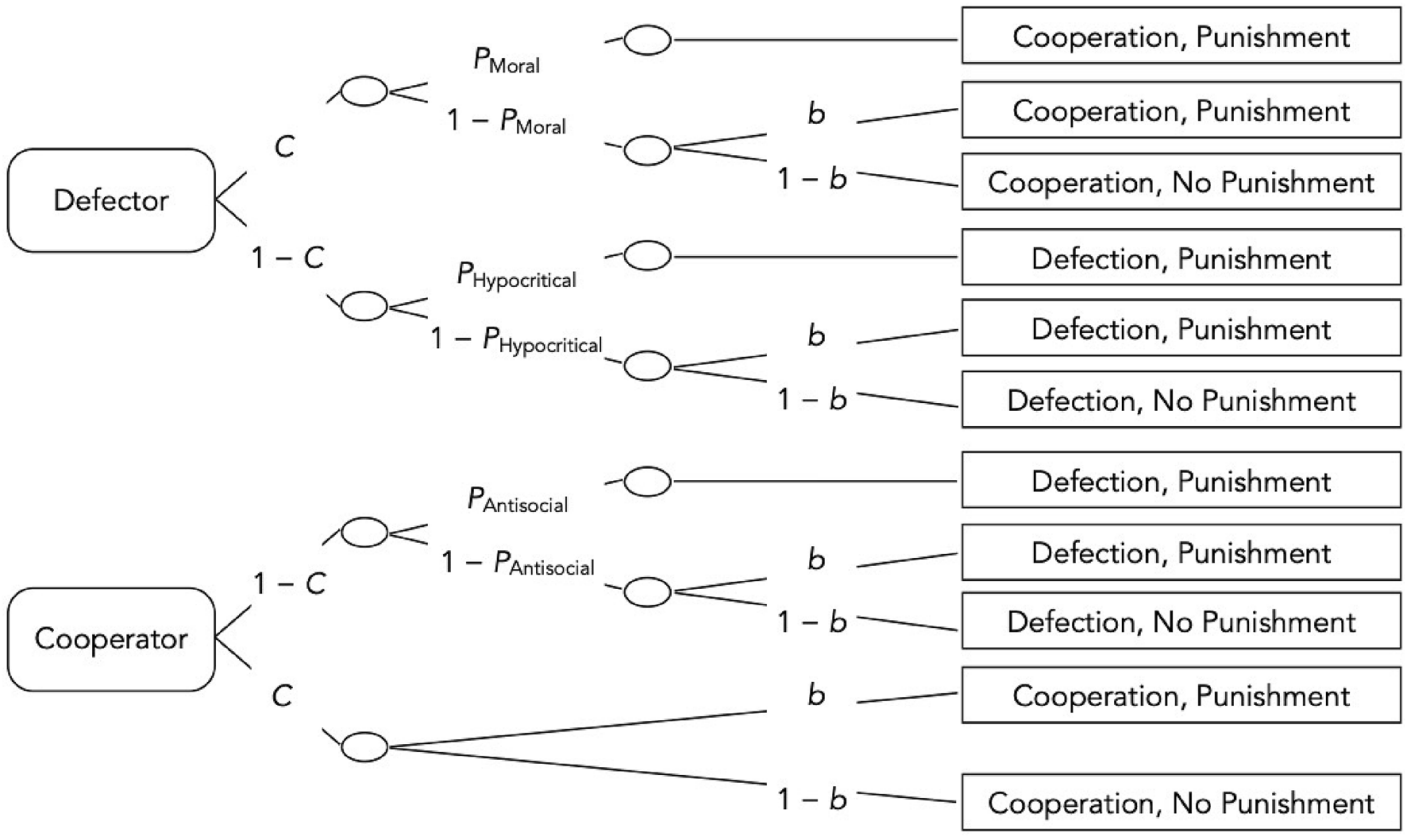
The multinomial cooperation-and-punishment model. The rounded rectangles on the left represent two types of partners that could be encountered in the Prisoner’s Dilemma game (defector or cooperator). The rectangles on the right represent the participant’s choices (cooperation or defection in the Prisoner’s Dilemma game; application or no application of punishment). The letters along the lines refer to the parameters of the model (*C* cooperation, *P*_*Moral*_ moral punishment, *P*_*Hypocritical*_ hypocritical punishment, *P*_*Antisocial*_ antisocial punishment, *b* punishment bias). To analyze the present results, two sets of the model trees shown here were needed, one for the no-load condition and one for the cognitive-load condition.

The participants’ choices in the Prisoner’s Dilemma game with punishment option were analyzed with the multinomial cooperation-and-punishment model (Fig. 3). Multinomial models serve to analyze how categorical data are determined by latent cognitive states and processes^54,55^. Historically, multinomial models have been used in genetic analyses to infer the underlying genotypic information from the observable phenotypic category frequencies^56^. For some decades, this class of models has also proven to be a useful tool for disentangling different component processes underlying moral judgements and behaviors and identifying different strategies underlying decision making (e.g.^57–61^). Computer programmes such as *multiTree*^62^ are used to estimate model parameters from observable behavior categories to perform statistical tests on these parameters. In the present application, the multinomial cooperation-and-punishment model serves to measure and disentangle cooperation, different types of punishment and a general punishment bias^42^. Rounded rectangles on the left side represent the two types of partners that were encountered in the game: defectors and cooperators. The rectangles on the right side represent the participant’s choices. The letters along the branches refer to the parameters of the model. These parameters represent probabilities that can vary between 0 and 1. In the Prisoner’s Dilemma game, the participant decides to cooperate with probability *C* or to defect with the complementary probability 1 − *C*. The model implies the assumption that the participant’s choice to cooperate or to defect is independent of the partner type determined by the partner’s behavior because the Prisoner’s Dilemma game is simultaneous which means that the partner’s behavior is revealed only after the participant has already made their decision.

If the participant decided to cooperate while the partner decided to defect, the participant may apply *moral punishment* with probability *P*_Moral_. However, if punishment is not caused by the moral choice to specifically punish the partner’s unilateral defection with the complementary probability 1 − *P*_Moral_, the participant may still show an unspecific punishment bias *b* to punish the partner irrespective of the outcome of the Prisoner’s Dilemma game. With probability 1 − *b* no punishment will be applied. After mutual defection, *hypocritical punishment* occurs with probability *P*_Hypocritical_. However, even if punishment does not occur as a consequence of the hypocritical choice to specifically punish mutual defection, punishment will be applied due to the general punishment bias with probability *b*. With the complementary probability 1 − *b*, mutual defection remains unpunished. If the participant decided to defect but the partner decided to cooperate, antisocial punishment occurs with probability *P*_Antisocial_. However, even if punishment does not occur as a consequence of the antisocial choice to specifically punish unilateral cooperation, punishment may occur as a consequence of a general punishment bias *b*. Mutual cooperation is special because there is little reason to assume that people should specifically punish mutual cooperation. Therefore, it has been proposed that any residual punishment in this condition is caused by an unspecific bias to punish the partner irrespective of the outcome of the Prisoner’s Dilemma game^42^. This allows us to estimate the *punishment bias b* which is parallel to how response bias is assessed in multinomial models in other domains (e.g.^63–65^). An advantage of this model is that the punishment parameters are represented as conditional probabilities so that punishment can be assessed independently of the level of cooperation in the Prisoner’s Dilemma game. This independence assumption is implied in the structure of the model and has also been empirically validated at a functional level in several studies that have demonstrated that the level of cooperation in the Prisoner’s Dilemma game can be manipulated without affecting the punishment parameters^40,41^. To analyze the present data, two sets of the model depicted in Fig. 3 are needed, one for the no-load condition and one for the cognitive-load condition. Hypotheses tests were performed by restricting the model further to incorporate the hypothesis that the parameters do not differ as a function of cognitive load. If this restriction caused a significant decrease in model fit, the hypothesis was rejected (cf.^55^). The model identifiability test, parameter estimations and goodness-of-fit tests were performed with *multiTree*^62^.

## Results

**Figure 4:**
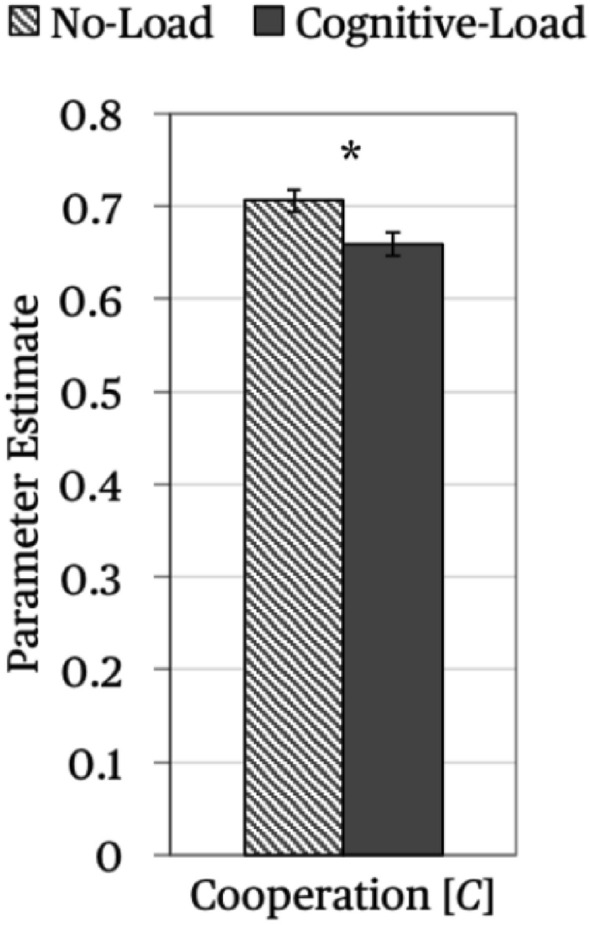
Parameter estimates of parameter *C* reflecting the probability that a participant cooperated in the no-load condition or the cognitive-load condition. The error bars represent standard errors. The asterisk highlights the significant difference between the no-load and the cognitive-load condition.

The base model (depicted in Fig. 3) fit the data well, *G*^2^ (2) = 2.35, *p* = 0.309. As depicted in Fig. 4, participants were more likely to cooperate in the no-load condition than in the cognitive-load condition, Δ*G*^2^ (1) = 7.14, *p* = 0.008, *w* = 0.05.

**Figure 5:**
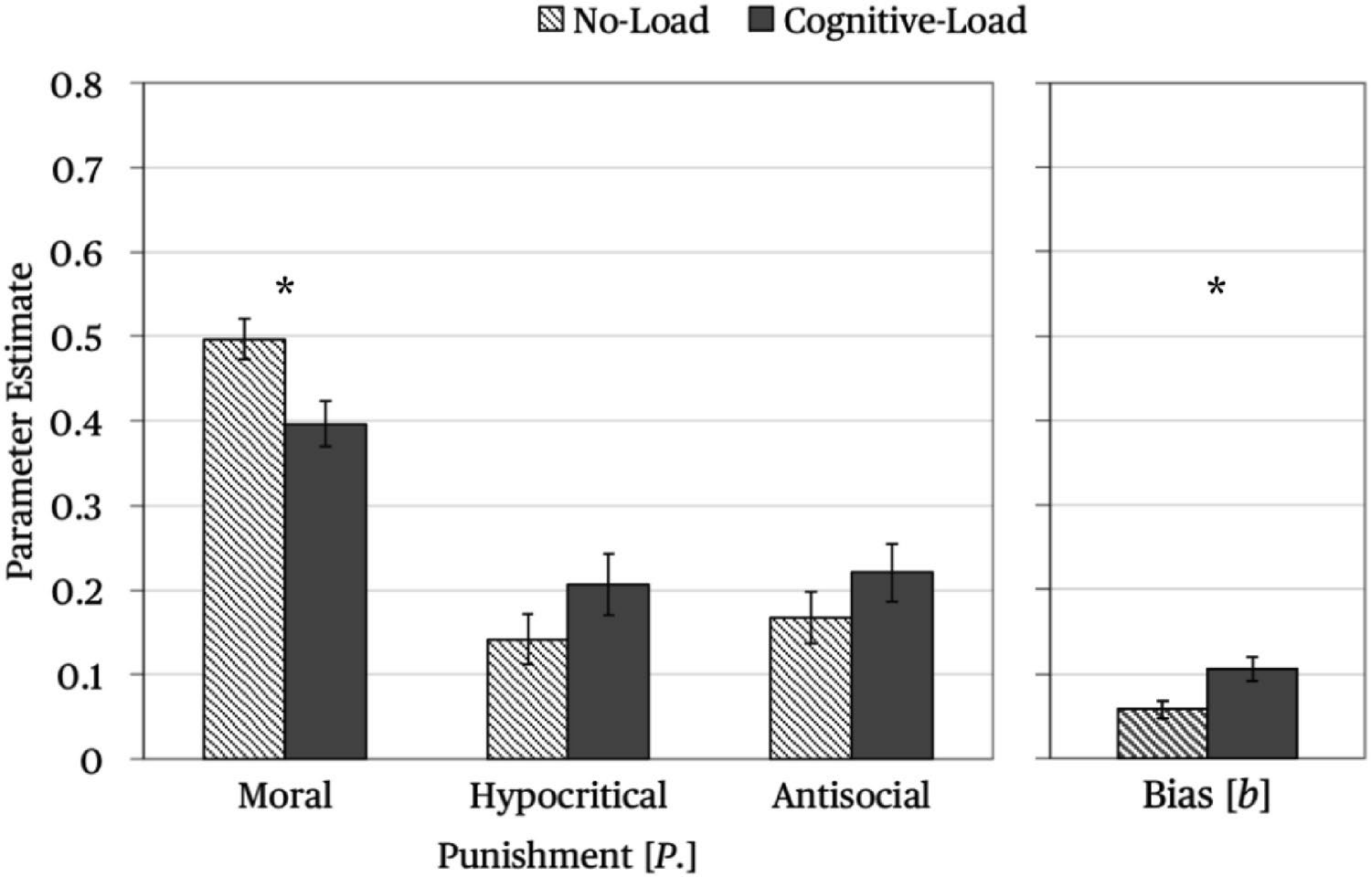
Left side: Parameter estimates of the punishment parameters reflecting moral punishment (*P*_Moral_), hypocritical punishment (*P*_Hypocritical_) and antisocial punishment (*P*_Antisocial_) in the no-load condition and the cognitive-load condition. Right side: Parameter estimates of the punishment-bias parameter (*b*), reflecting the general tendency to punish the partners irrespective of the outcome of the Prisoner’s Dilemma game. The error bars represent standard errors. The asterisks highlight significant differences between the no-load condition and the cognitive-load condition.

The parameters representing moral, hypocritical and antisocial punishment are shown in the left panel of Fig. 5. The punishment bias is shown in the right panel of Fig. 5. Moral punishment occurred more often in the no-load condition than in the cognitive-load condition, Δ*G*^2^ (1) = 7.71, *p* = 0.006, *w* = 0.05. By contrast, neither hypocritical punishment, Δ*G*^2^ (1) = 1.90, *p* = 0.169, *w* = 0.03, nor antisocial punishment, Δ*G*^2^ (1) = 1.30, *p* = 0.253, *w* = 0.02, differed as a function of the dual-task manipulation. However, participants showed an increased punishment bias in the cognitive-load condition in comparison to the no-load condition, Δ*G*^2^ (1) = 7.51, *p* = 0.006, *w* = 0.05, suggesting that punishment was less purposefully applied when cognitive resources were decreased.

A limitation of the model-based analysis reported above is that it is only concerned with the probability of punishment but not with the height of punishment. Therefore, we performed a supplementary analysis of the height of punishment. If participants decided to punish, they spent 3.7 cents (*SD* = 2.2) on average to deduce 37 cents from their partner’s account. The height of punishment did not differ between the cognitive-load condition and the no-load condition, *F* (1,138) = 0.17, *p* = 0.685. This conclusion holds when analyzed separately for the different combinations of cooperative and non-cooperative behaviors of participants and partners (all *p*’s > 0.361). Individual-level data on the frequencies of punishment and the sums invested into punishment are available at the OSF project page (see data-availability section) to allow other researchers to perform alternative analyses of the data.

## Discussion

A hotly debated topic is whether moral behaviors such as cooperation and punishment are intuitive or deliberate. Dual process theories^3^ imply that human behavior is based on two cognitive systems. Behavior based on the Type-I system is intuitive, effortless and automatic, whereas behavior based on the Type-II system is deliberate, effortful and controlled^23^. The intuitive-morality view predicts that cooperation in one-shot interactions is intuitive whereas defection is deliberate^5^. Thus, cooperation in one-shot interactions should increase when cognitive deliberation is suppressed by a cognitive-load manipulation. By contrast, the deliberate-morality view^24^ predicts that choosing the moral option requires deliberate control of selfish intuitions. However, the empirical evidence is mixed^11,66,67^, with some studies showing that cooperation increases under cognitive load^8,9,11^ while others show decreased cooperation under cognitive load^28^. Therefore, it is likely that the influence of cognitive load on cooperation depends on the situation^2,12^. Here, we used a previously established and validated cognitive-load task^10^ to reduce the cognitive resources that were available during a Prisoner’s Dilemma game with a punishment option. As it turns out, the cooperation rate was lower in the cognitive-load condition than in the no-load condition. While this result at first glance seems to favor the deliberate-morality view, it is important to keep in mind that the participant’s unilateral defection was always punished by the partners in the Prisoner’s Dilemma game. The fact that participants were punished for unilateral defection changes the incentive structure of the Prisoner’s Dilemma game by providing an incentive for cooperation, which renders cooperation the rational choice. When morality and rationality are aligned, the availability of cognitive resources can be expected to increase cooperation (cf.^5^). An interesting possibility is that participants were defecting more under cognitive load because it is a simple strategy that is only based on the participant’s immediate payoffs while cooperation may be more demanding as it requires the participants to engage in second-order reasoning about what the partner will do based on the previous experiences in the Prisoner’s Dilemma game.

To explore whether participants increased their cooperation rate over the course of the experiment to adapt to the moral punishment by the partners, we compared cooperation rates between the first half of the experiment and the second half of the experiment. Interestingly, the cooperation rate in the Prisoner’s Dilemma game increased from the first half to the second half of the experiment in the no-load condition, Δ*G*^2^ (1) = 5.79, *p* = 0.016, *w* = 0.04, but there was no corresponding increase of the rate of cooperation in the cognitive-load condition, Δ*G*^2^ (1) = 1.67, *p* = 0.197, *w* = 0.02. This suggests that participants may have been less likely to increase their cooperation rates in response to the moral punishment by the partners in the cognitive-load condition in comparison to the no-load condition.

The main purpose of the present study was to test whether moral punishment increases or decreases under cognitive load. Moral punishment can be seen as a form of second-order cooperation because it is collectively beneficial but individually costly. This dilemma results in a conflict between the moral motive to punish the partner for the violation of the fairness norm and the selfish motive to avoid personal costs. According to the intuitive-morality view, it is thus to be expected that moral punishment is intuitive and that participants should shy away from the costs of punishment when they have the cognitive capacity to make economically rational decisions. While some studies in the Ultimatum-game paradigm have been interpreted to be consistent with this view^30,31^, neuroimaging studies consistently found brain areas associated with cognitive control to be involved in costly punishment^2^. The latter findings suggest that costly punishment is deliberate rather than intuitive^38^. In line with this view, the present study found that moral punishment decreased under cognitive load.

An interesting observation is that cognitive load did not decrease all types of punishment. Specifically, hypocritical and antisocial punishment did not decrease under cognitive load. Instead, there was even a descriptive (but non-significant) tendency for hypocritical and antisocial punishment to be enhanced under cognitive load. Given that these forms of punishment are often attributed to spite and aggression^43,44^, it seems possible to conclude that costly spiteful and aggressive behaviors are not favored by cognitive deliberation. Furthermore, cognitive load induced an unspecific bias to punish the partners regardless of the outcome of the Prisoner’s Dilemma game, suggesting that punishment was less purposefully applied when the participants were distracted by the continuous-tone classification task which is in line with the idea that the reduction of cognitive resources might induce more random behavior^34^. Given that less purposefully applied punishment may have disruptive effects on social interactions^68^, this finding underlines the general point that the lack of cognitive resources does not always favor the social good.

While the present findings thus support the deliberate-morality view, further research is necessary before broad generalizations about moral punishment are made. Even though moral punishment was applied in a deliberative and reflective manner in the present paradigm, it seems possible or even likely that other forms of costly punishment (e.g., more violent forms of retributions) have to be classified as intuitive and may occur with a higher probability when cognitive resources are decreased. At the present time, it seems to be too broad of a generalization to conclude that punitive decisions are always favored by the availability of cognitive resources. Before such broad conclusions are drawn, future studies should examine how cognitive load affects costly punishment across a range of different situations. Furthermore, future studies could explore whether the effect of cognitive load on moral punishment might be moderated by inter-individual differences in punitive attitudes. Another aspect that deserves some discussion is that participants interacted with computer-controlled partners in the present study. This is a typical approach in Experimental Psychology in which the goal is to identify the factors that determine the individual’s behavior so that the behavior of the partners is seen as an extraneous influence on the participant’s behavior that needs to be experimentally controlled (e.g.^69–75^). This approach contrasts with that of Experimental Economics in which the focus lies on the interactions of dyads or groups of human participants. However, it seems noticeable that the different types of punishment were shown with similar probabilities as in previous studies examining interactions among human partners (e.g.^43,44^). These similarities suggest that the paradigm used here taps into the same mechanisms. However, this assumption remains to be tested in future studies. Beyond that, it seems sensible to discuss possible effects of the fact that the partners in the present study were programmed to morally punish the participants. Specifically, participants might have learned from their partners, during the course of the experiment, that moral punishment is the appropriate behavior. A priori this seems unlikely given that studies using the same paradigm have revealed high levels of moral punishment regardless of whether participants experienced moral punishment by their partners or not^40,41^. We nevertheless conducted a supplementary analysis of the present data which revealed that moral punishment did not differ between the first and the second half of the experiment in both the no-load condition, Δ*G*^2^ (1) = 0.44, *p* = 0.506, *w* = 0.01, and the cognitive-load condition, Δ*G*^2^ (1) = 1.16, *p* = 0.282, *w* = 0.02. The other types of punishment did not differ between the first and the second half of the experiment either, in both the no-load condition (hypocritical punishment: Δ*G*^2^ (1) = 0.11, *p* = 0.744, *w* < 0.01; antisocial punishment: Δ*G*^2^ (1) = 0.57, *p* = 0.450, *w* = 0.01) and the cognitive-load condition (hypocritical punishment: Δ*G*^2^ (1) = 0.02, *p* = 0.875, *w* < 0.01; antisocial punishment: Δ*G*^2^ (1) = 1.75, *p* = 0.186, *w* = 0.02). The bias parameter, by contrast, was decreased in the second compared to the first half of the experiment in the no-load condition (Δ*G*^2^ (1) = 7.82, *p* = 0.005, *w* = 0.05) but not in the cognitive-load condition (Δ*G*^2^ (1) = 0.32, *p* = 0.574, *w* = 0.01), suggesting that punishment was more purposefully applied in the second half of the experiment when cognitive resources were not diverted by a distractor task.

## Summary and conclusions

To conclude, we found a negative effect of cognitive load on cooperation and moral punishment in a Prisoner’s Dilemma game with punishment option. Under limited cognitive resources, cooperation and moral punishment declined. Punishment was also applied less purposefully under cognitive load. Taken together, these findings suggest that the dominant view that cognitive deliberation decreases moral decisions in one-shot interactions is too simplistic. Some moral decisions seem to benefit from cognitive deliberation and are thus reduced under cognitive load.

## Data Availability

The data analyzed in the current study are available at https://osf.io/hq53g.
